# Electron microscopy reveals saturated fatty acid-induced membrane defects in AdipoR2-depleted cells

**DOI:** 10.1186/s12944-025-02804-2

**Published:** 2025-12-01

**Authors:** Dimitra Panagaki, Mario Ruiz, Ranjan Devkota, Johanna L. Höög, Richard Neutze, Marc Pilon

**Affiliations:** Department of Chemistry and Molecular Biology, University of Gothenburg, Box 115, Gothenburg, S-405 30 Sweden

**Keywords:** Lipids, Phospholipids, Mitochondria, Lipotoxicity, Phospholipids/trafficking, Membrane fluidity, AdipoR2, Palmitate, Electron microscopy

## Abstract

**Background:**

Maintaining a proper balance between saturated and unsaturated fatty acids in membrane phospholipids is essential for normal cellular function. The evolutionarily conserved transmembrane protein AdipoR2 plays a central role in this homeostatic process. While the detrimental effects of saturated fatty acids on cells have been previously documented, the associated ultrastructural changes remain less investigated.

**Methods:**

Here, we used transmission electron microscopy to study the consequences of silencing AdipoR2 in the presence or absence of fatty acid supplements.

**Results:**

We found that exposure of human cells to palmitic acid (PA)—the most abundant saturated fatty acid in the human body—disrupts the ultrastructure of cytoplasmic membranes and mitochondrial cristae. PA exposure also induces distinctive blebbing between the inner and outer membranes of the nuclear envelope. These membrane abnormalities are exacerbated by AdipoR2 silencing and are partially prevented by supplementation with oleic acid (OA), an unsaturated fatty acid. Furthermore, we observed ectopic localization of the mitophagy marker PINK1 and the fatty acid metabolism enzyme ACSL1 to closely apposed ER membranes, a structure that forms exclusively in PA-treated cells.

**Conclusions:**

Together, these findings reveal that exogenous PA triggers significant membrane defects, worsened in the absence of AdipoR2, and alters protein distribution within the cell.

**Supplementary Information:**

The online version contains supplementary material available at 10.1186/s12944-025-02804-2.

## Background

Cells adaptively adjust the lipid composition of their membranes in response to different environmental conditions [1, 2]. Conversely, membrane lipid homeostasis defects can irreversibly affect important cellular processes such as protein trafficking, vesicle formation and receptor signaling [3–5]. Several molecular mechanisms exist that sense and remedy imbalances in the fatty acid composition of the membranes [6]. Adiponectin receptor 2 (AdipoR2), best known for its role in preventing metabolic syndrome [7–10], is an evolutionarily conserved protein critical for cell membrane homeostasis [11–16]. AdipoR2 is activated by membrane rigidification, for example when there is an excess of saturated fatty acids (SFAs) in phospholipids. Once activated, a ceramidase activity intrinsic to AdipoR2 [7, 17, 18] yields a sphingosine that, upon phosphorylation, converts to sphingosine 1-phosphate that acts as a signal to promote fatty acid desaturase expression and ultimately restore membrane fluidity by increasing its unsaturated fatty acid (UFA) content [19].

When cells are challenged with high concentrations of exogenous SFAs, their membranes become more rigid, increasing both endoplasmic reticulum (ER) stress and apoptosis [1, 20, 21]. These SFA-associated defects are exacerbated in the absence of a functional AdipoR2: cells lacking AdipoR2 have a slower growth rate in basal conditions and this worsens in the presence of the SFA palmitic acid (PA; C16:0), which also causes pronounced transcriptome changes similar to those in SREBP-deficient cells [13], accompanied by the formation of tightly packed rigid ER membranes [19, 22]. A potent way of alleviating these PA-induced defects is the supplementation of a UFA such as the monounsaturated oleic acid (OA, C18:1) [11, 23]. Most mouse tissues express both AdipoR1 and AdipoR2, which are at least partially redundant since the double mutant, but neither single mutant, is embryonic lethal [19, 24]. However, only AdipoR2 is expressed in testis where the AdipoR2 knockout shows pronounced nuclear membrane rigidification that causes membrane buckling and impaired chromosome synapsis during spermatogenesis, resulting in male sterility [25].

Despite numerous studies documenting the deleterious cellular and physiological effects of excess membrane SFAs, the consequences on intracellular membrane ultrastructural morphology have been described in only a few studies. Recent electron tomography revealed that increasing the level of endogenously produced SFAs in budding yeast (*Saccharomyces cerevisiae*) changed round nuclei to irregular polygons, and ER membranes to straight lines with edges shaped like blisters [26]. Electron microscopy analysis of human liver cancer (HepG2) cells exposed to PA showed enlarged mitochondria, with granular matrix, dense matrix granules and reduced numbers of cristae [27]. Confusingly, a separate study also performed transmission electron microscopy of HepG2 cells and noted no morphological changes to mitochondria [28]. Instead, cells exposed to palmitate had “deranged ER morphology”, with large cracks in the electron microscopy preparation following the ER cisternae.; these cracks were partially prevented by the addition of OA.

To resolve and quantify the morphological effects that altered lipid composition can have on intracellular membranes, we applied electron microscopy of high-pressure frozen human embryonic kidney cells (HEK293) and near-haploid HAP1 cells to examine and quantify the severity of membrane deformations in control and AdipoR2-deficient cells challenged with PA. While exploring functional consequences of the membrane defects, we also found that the mitophagy marker PINK1 and the enzyme ACSL1 (involved in fatty acid metabolism) have altered distributions among membrane compartments in PA-challenged cells, and that co-cultivation with OA prevents PA-induced morphological defects.

## Methods

### Cell culture

HEK293 cells were obtained from the American Type Culture Collection (ATCC) and grown in DMEM containing L-glutamine, 25 mM HEPES and supplemented with 1% penicillin and streptomycin, 1% MEM NEAA (non-essential amino acids), and 10% FBS (fetal bovine serum), all from Life Technologies) at 37 °C in a water-humidified 5% CO_2_ incubator. Cells were subcultured twice a week at 90% confluence. TrypLE Express reagent (Gibco) was used to detach the cells. All cell types were cultivated on treated plastic flasks and multiwell plates (Nunc). Parental (“wild-type”) and AdipoR2-KO HAP1 cells were obtained from Horizon. AdipoR2-KO cells were CRISPR/Cas9-edited (5 bp deletion in exon 3: HZGHC003913c002). HAP1 was grown in IMDM media containing L-glutamine, 25 mM HEPES and supplemented with 1% penicillin and streptomycin, 1% MEM NEAA and 10% FBS. HAP1 were subcultured twice a week at 75–80% confluency.

### Fatty Acid (FA) treatment

PA and OA were dissolved in dimethyl sulfoxide (DMSO, Sigma-Aldrich). The fatty acids were mixed in 0.5% fatty acid-free bovine serum albumin (BSA; Sigma-Aldrich) in serum-free medium for 15 min at room temperature. The resulting molecular ratios of BSA to PA were 1:5.3 in experiments that used 400 µM PA, 1:2.65 in experiments that used 200 µM PA etc. Cells were then incubated in serum-free media containing BSA alone (basal media) or BSA/FA conjugates for 18 h before analysis.

### siRNA treatment

Non-targeting (NT) D-001810–10 and AdipoR2 J-007801–10 siRNAs were obtained from Dharmacon and used in a previously validated protocol for HEK293 cells [16, 29]. Briefly, transfection of 25 nM siRNA in complete medium using Viromer Blue was carried out according to the manufacturer’s instructions 1X (Lipocalyx). The knockdown expression of the AdipoR2 gene to < 25% of control levels was verified by quantitative PCR (qPCR) as in previous studies [16, 29].

### Mitochondrial quantification

For the mitochondrial quantifications of the electron micrographs, 100 randomly selected mitochondria per treated group, were selected and modeled with the use of the IMOD software package [30]. Mitochondria were modeled as closed contours and the area and perimeter were calculated with the ‘imodinfo’ command line. The long and short diameters were measured with the use of the 3dmod measuring tool. The aspect ratio was calculated by dividing the long to the short diameter and the circularity was based on the following mathematical equation:

$$\text{Circularity} = 4^{*}\!3, 14^{*}\! (\text{area/perimeter}^{\wedge}2)$$

### High-pressure freezing of cells for electron microscopy and tomography

Cells were concentrated by mild centrifugation into sealed 200 µl pipet tips as described before [31], loaded into aluminum specimen carriers and then high-pressure frozen in a Wohlwend Compact 3 (M. Wohlwend GmbH, Sennwald, Switzerland). The growing media of the cells was used as a cryoprotectant. A short freeze substitution protocol was applied, using 2% uranyl acetate (UA) for three hours prior to the acetone washes [32, 33]. Samples were then embedded in HM20 resin and polymerized under UV light. For electron microscopy, samples were sectioned in 70 nm thin sections and placed on copper slot grids. Double side staining with 15 nm colloidal gold fiducial particles was performed on the grids before imaging. On sections, contrast staining of the samples was performed using 2% UA and Reynold’s lead citrate [34].

### Immuno-electron microscopy

For the immuno-labeling experiment, the same high-pressure frozen samples embedded in HM20 resin were used. Grids with 70 nm thick sections were fixed in 1% paraformaldehyde (PFA) in PBS for 10 min and blocked with 0.1% fish skin gelatin and 0.8% BSA in PBS for 1 h. For primary antibody labeling, samples were incubated overnight at 4 °C with a PINK-1 rabbit antibody (mAb #6946, Cell Signaling Technology), Calnexin rabbit antibody (C5C9, Cell Signaling Technology), ACSL1 rabbit antibody (D2H5, Cell Signaling Technology) or SREBP1 mouse antibody (2A4, Santa Cruz) followed by a one-hour incubation with a secondary goat-anti-rabbit or goat-anti-mouse 15 nm gold antibody (Electron Microscopy Sciences). 2.5% glutaraldehyde was applied on sections for 1 h followed by contrast staining as described above.

### Statistics

For the deformed mitochondria, the percentage of deformed mitochondria per cell section was calculated and plotted. A non-parametric One-way ANOVA (Kruskal-Wallis test) followed by a Dunn’s multiple comparisons test, was performed for comparisons between the treated groups. For the total percentages of the cytoplasmic membrane defects and the nuclear envelope buddings, a Fisher’s exact test was performed between sample pairs. Asterisks are used in the figures to indicate various degrees of significance, as following: *p < 0.05; **p < 0.01; ***p < 0.001; and ****p < 0.0001.

## Results

### PA induces distinct membrane deformations in HEK293 cells

To define PA-induced defects in the intracellular structure and morphology of human cells, we treated HEK293 cells with three different concentrations of PA (100, 200 and 400 µM) then examined them using transmission electron microscopy where 30–40 cell sections from each treatment were randomly selected and imaged. We found that three distinct cellular compartments were most affected in the PA-challenged cells.

**Fig. 1:**
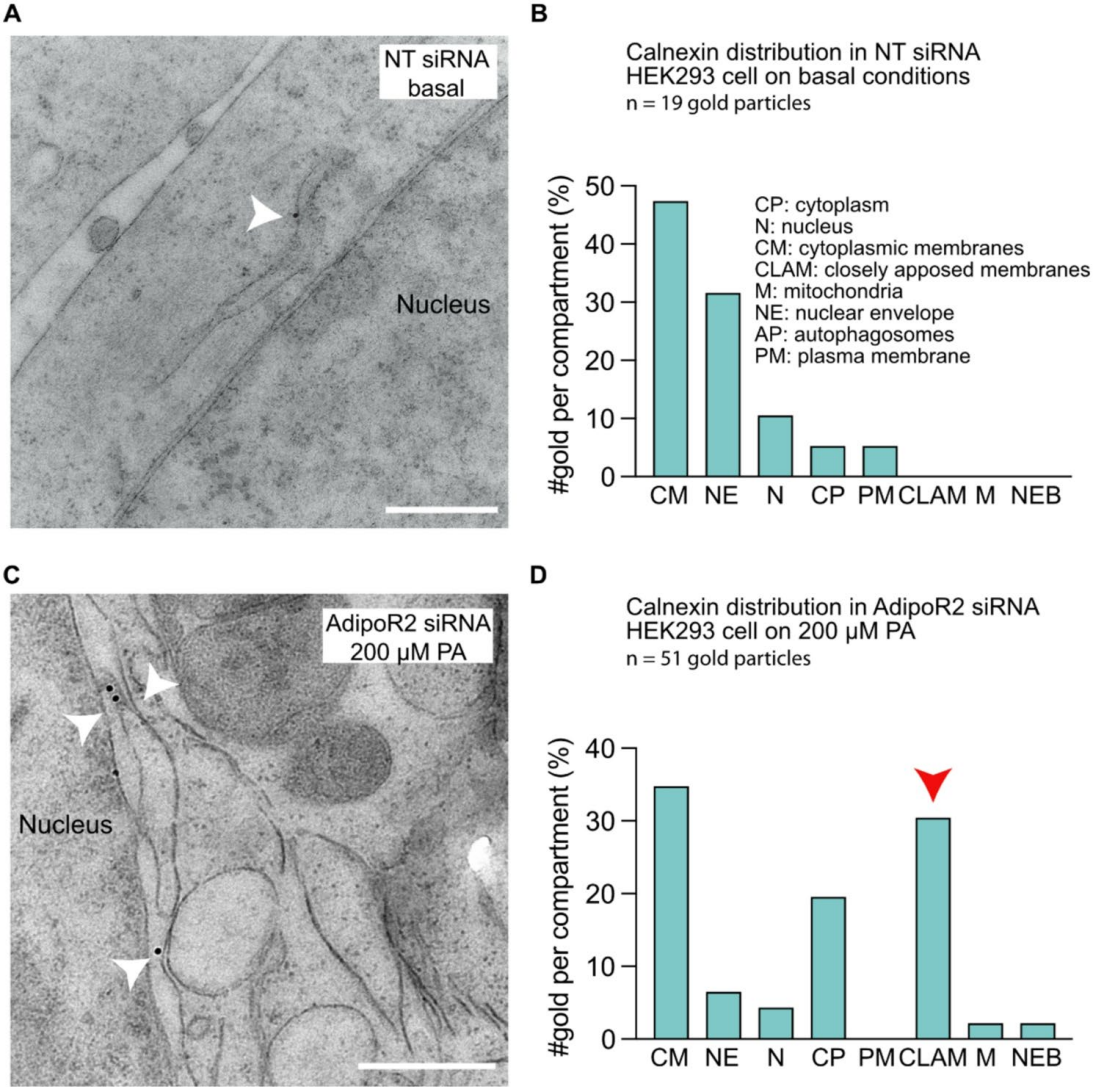
The ER marker calnexin is localized at the closely apposed membranes. An immuno-EM assay was performed in NT siRNA cells on basal conditions and AdipoR2 siRNA cells on PA for the localization of the ER-marker calnexin. **(A)** Representative electron micrograph showing a gold particle (white arrowhead) bound to normal ER in an NT siRNA cell on basal conditions. **(B)** Number of gold particles found in 8 different cellular compartments. The majority of the gold was localized at the cytoplasmic membranes of the cells (ER). **(C)** Representative electron micrograph showing gold particles associated with the closely apposed membranes (white arrowheads) as well as a connection to the closely apposed membranes with the nuclear envelope in PA-treated AdipoR2 siRNA cells. **(D)** Calnexin distribution in 8 different cellular compartments in AdipoR2 siRNA cells on PA. The amount of gold particles bound to closely apposed membranes (emphasized by the arrowhead) was similar to the amount found on cytoplasmic membranes (ER) and higher than any other examined compartment. Abbreviations: CM; cytoplasmic membranes, NE; nuclear envelope, N; nucleus, CP; cytoplasm, PM; plasma membrane, CLAM; closely apposed membranes, M; mitochondria, NEB; nuclear envelope buds
(ii) A second affected compartment is the nuclear envelope. In the PA-treated cells the inner nuclear membrane (INM) and outer nuclear membrane (ONM) were often separated from each other by heterogeneous buddings of different sizes and morphologies located between the two membranes (Fig. 2C-D). A similar phenotype has been reported in cells exposed to heat or other stressors and these structures were then referred to as “nuclear envelope buddings” (NEBs) [36], a terminology that we will adopt here.

**Fig. 2:**
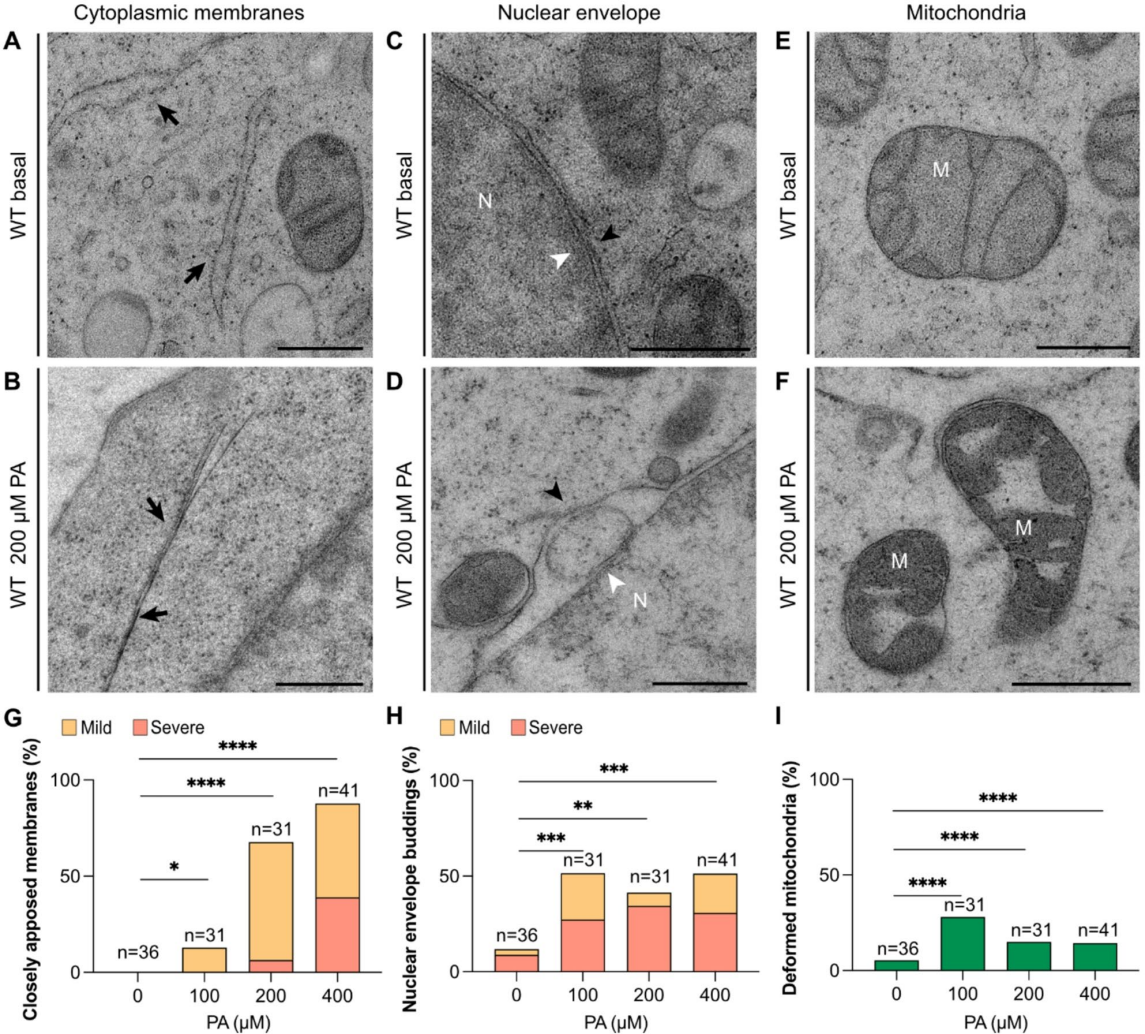
Overview of PA-induced cellular deformations in HEK293 cells. HEK293 cells were grown under basal, 100, 200, and 400 µM of PA. Electron micrographs depicting morphologically normal ER **(A)** and closely apposed membranes **(B)**, normal nuclear envelope membranes **(C)** and NEB **(D)**, and normal mitochondria **(E)** and deformed mitochondria **(F).** Black arrows in panels A and B indicate ER membranes. Black arrowheads in panels C and D point at the outer nuclear membrane whereas white arrowheads point at the inner nuclear membrane. **(G-I)** HEK293 cells were grown at 0 µM (basal), 100 µM, 200 µM and 400 µM PA and random images of at least 31–41 cell sections per condition were taken and quantified for each cellular compartment of interest. The closely apposed membranes and NEB phenotypes were further categorized as “mild” when less than three instances were observed in one cell section or as “severe” when three or more instances were observed. Abbreviations: N; nucleus, M; mitochondria. Scale bars: 500 nm. *p < 0.05; **p < 0.01; ***p < 0.001; and ****p < 0.0001
(iii) Thirdly, some mitochondria displayed a characteristic phenotype with cristae that were swelled, deformed and disorganized compared to non-treated cells (Fig. 2E-F). This is particularly interesting given the previously reported mitochondrial dysfunction in AdipoR2 deficient cells [13].

(i) An initial experiment showed that cytoplasmic membranes in PA-treated cells form straight structures, dubbed “closely apposed membranes”, that are positive for the ER marker Calnexin **(**Fig. 1**)**. Subsequent dose-response experiments showed that these closely apposed membranes appear more frequently with increasing PA doses (Fig. 2A-B). To establish an ER scoring system for the present study, we measured the width of the ER lumen in cells treated with basal media, PA or OA and used the resulting quantification as benchmarks for classification of ER membranes as “normal” (~ 90 nm; as in basal media), “closely apposed” (< 30 nm; as in 400 µM PA) or “swollen” (>200 nm; as in 100 µM OA) (Figure S1). These classification criteria align well with published studies. In particular, the diameter of tubular ER is typically ~ 60–90 nm, with the exception of a narrow ER class found in neuronal axons that has a diameter of 20 to 30 nm [35]. The present study will make extensive use of siRNA, and we also verified that a control nontarget siRNA treatment had no significant effect on the ER (Figure S1).

The response of the three PA-induced phenotypes was quantified for the 0–400 µM PA range (Fig. 2G-I), which revealed a dose-dependent effect for the closely apposed membrane phenotype (present in 90% of cells treated with 400 µM PA). The NEBs and mitochondrial defects were induced by 100 µM PA but did not increase with higher doses. Note that the PA concentrations tested do not exceed physiological plasma concentrations, which range from 300 µM to 3 000 µM or more [37, 38].

### Depletion of AdipoR2 enhances PA-induced membrane deformations

**Fig. 3:**
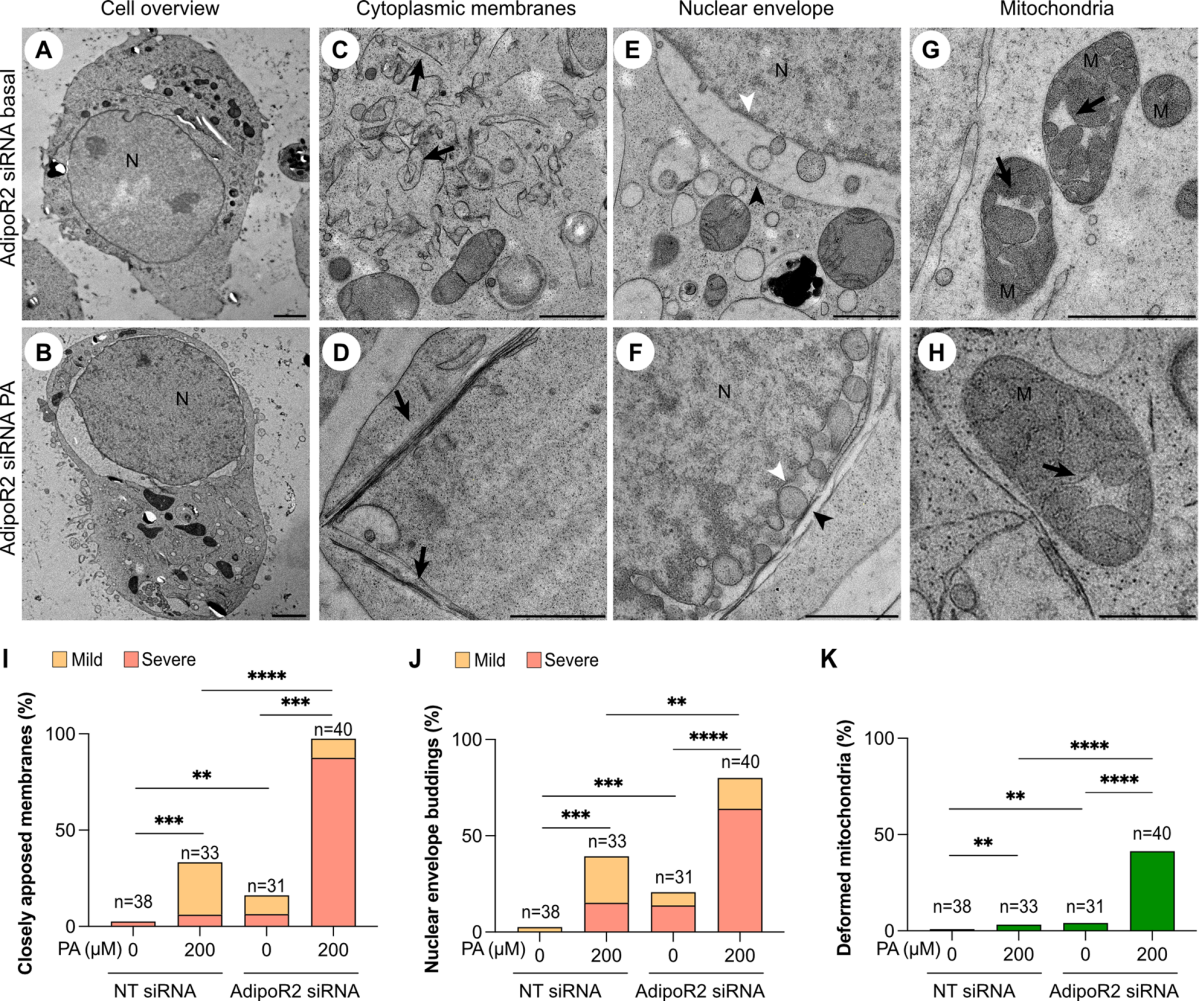
AdipoR2-defficient cells have more PA-induced cellular deformations.** (A-B)** Electron micrograph overviews of an AdipoR2 siRNA-treated cell grown in basal conditions or in presence of 200 µM PA, respectively. **(C-H)** Electron micrographs of the indicated membrane structures in AdipoR2 siRNA-treated cell grown on basal conditions or in presence of 200 µM PA. Black arrows in panels C and D point at closely apposed membranes. Black arrowheads in panels E and F point at the outer nuclear membrane whereas white arrowheads point at the inner nuclear membrane. Black arrows in panels G and H point at the deformed cristae of the mitochondria. **(I-K)** HEK293 cells treated either with a non-targeted (NT, control) or an AdipoR2 siRNA were grown either on 0 µM (basal) or 200 µM PA and several randomly chosen micrographs were acquired and quantified for the frequency of three different morphological deformations. The AdipoR2 siRNA cells on PA showed the highest frequency for all studied phenotypes. The closely apposed membranes and NEB phenotypes were further categorized as “mild” when less than three instances were observed in one cell section or as “severe” when three or more instances were observed. At least 30–40 cell sections per condition were analyzed. Abbreviations: N; nucleus, M; mitochondria. Scale bars: A-B; 5 μm, D-H: 1 μm. *p < 0.05; **p < 0.01; ***p < 0.001; and ****p < 0.0001

**Fig. 4:**
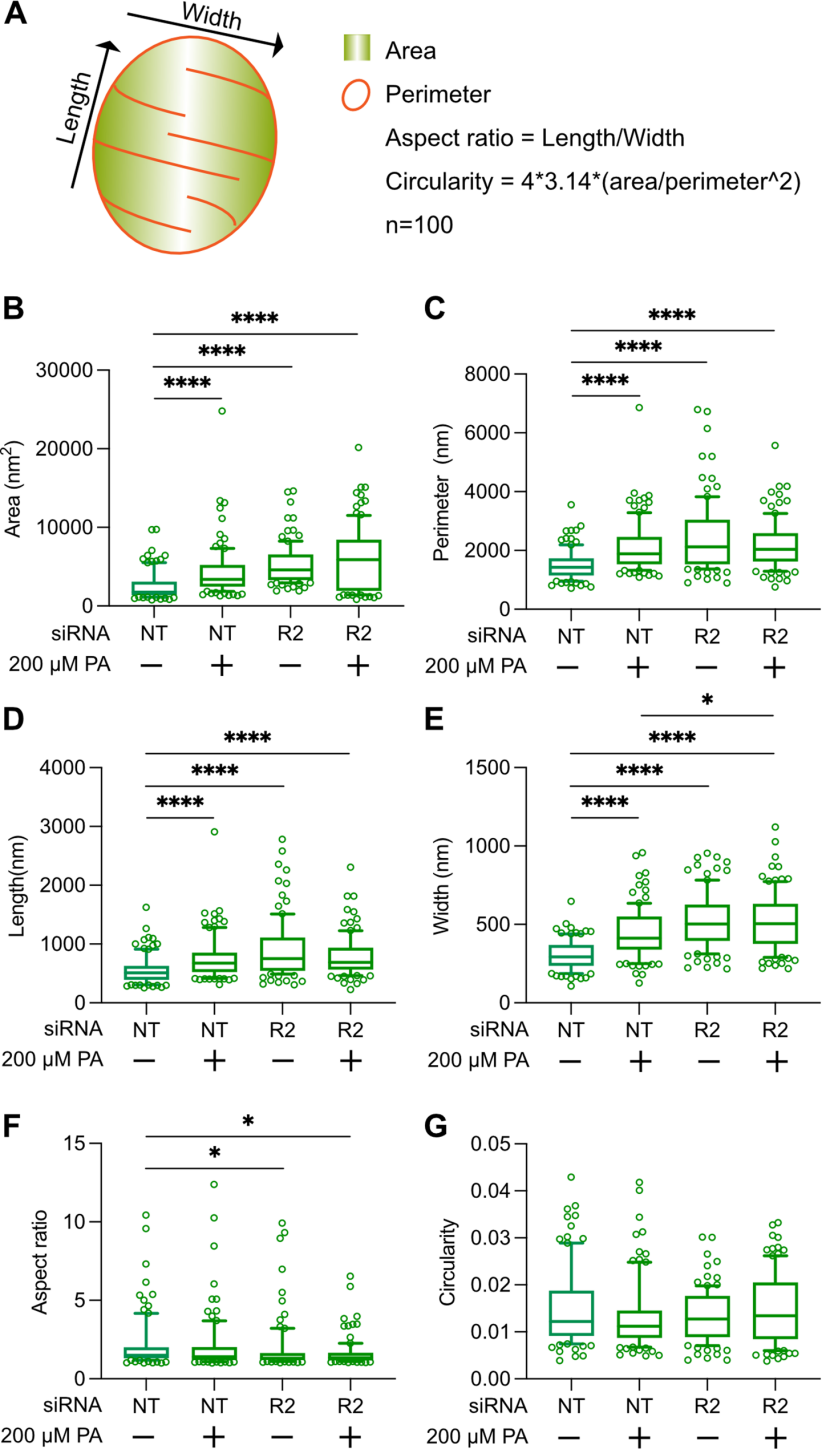
AdipoR2-deficient cells have larger mitochondria. Mitochondria from non-targeted and AdipoR2 siRNA-treated HEK293 cells grown on 0 µM (basal) or 200 µM PA and electron micrographs depicting 100 randomly acquired mitochondria were modeled as closed contours in the IMOD software program and the command *imodinfo* was used to calculate area and perimeter. The measurement tool was used to determine the long and short diameters. **(A)** Model depicting mitochondrial measurements and the mathematical formulas for the calculation of the aspect ratio and circularity. **(B-G)** Graphs with the calculated parameters per treated conditions showing that PA- and AdipoR2 siRNA-treated cells have larger mitochondria in comparison to the control groups. *p < 0.05; ***p < 0.001; and ****p < 0.0001

AdipoR2-deficient cells have decreased membrane fluidity, especially when exposed to exogenous SFAs [11, 16, 19, 29, 39]. Here, AdipoR2 siRNA- and control non-targeted siRNA-treated HEK293 cells, cultivated in basal media or in the presence of 200 µM PA, were prepared for electron microscopy. From each treatment group, 30 to 50 cell sections were randomly selected and imaged. All three membrane deformations observed in control HEK293 cells challenged with PA were again observed in this experiment (Fig. 3A-H): AdipoR2 siRNA cells exhibited membrane deformations even in basal media but, as expected, the frequency and severity of all three types of membrane defects were highest in the AdipoR2 siRNA-treated cells challenged with PA where a majority contained these deformed membranes (Fig. 3I-K). We also found that the mitochondria cristae defects in PA or AdipoR2 siRNA-treated cells were accompanied by an overall enlargement of the mitochondria while preserving their overall proportions (Fig. 4), suggesting defects in mitochondria homeostatic processes such as fusion/fission or mitophagy.

We conclude that AdipoR2 contributes to membrane homeostasis even in unchallenged conditions but is particularly important in membrane-rigidifying conditions (i.e. in the presence of PA) where it normally prevents the close apposition of ER membranes, formation of nuclear envelop buds and mitochondrial enlargement and cristae swelling.

### Co-cultivation with OA prevents PA-induced morphological deformations

**Fig. 5:**
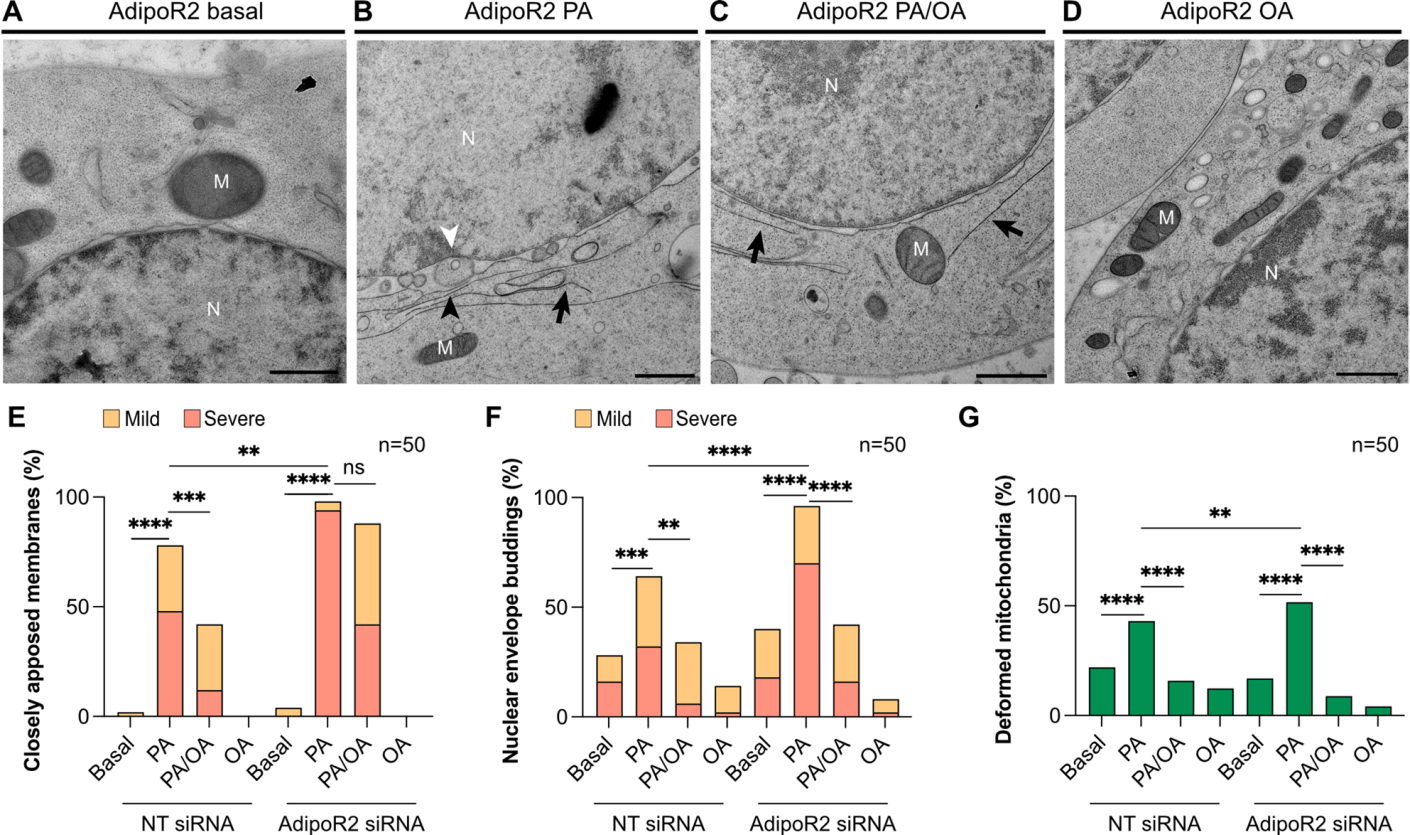
PA-induced deformations are prevented by co-cultivation with OA.** (A-D)** Non-targeted or AdipoR2 siRNA-treated HEK293 cells were grown with 0 µM (basal) or 200 µM of PA as well as 200 µM/100 µM of PA/OA together or 100 µM of OA. These electron micrographs represent the morphological differences in the ER, nuclear envelope and mitochondria per condition. Black arrows in panels **B** and **C** point at the closely apposed membranes. The black arrowhead in panel **B** points at the outer nuclear membrane whereas the white arrowhead points at the inner membrane with visible nuclear buds between the two membranes. **(E-G)** Random images of at least 50 cell sections per condition were acquired and quantified for the frequency of the observed deformations. Addition of OA reduced the frequency of all but one morphological deformation. For the closely apposed membranes of the AdipoR2 siRNA cells, addition of OA did not significantly alter the frequency but did reduce the severity of the phenotype. The closely apposed membranes and NEB phenotypes were further categorized as “mild” when less than three instances were observed in one cell section or as “severe” when three or more instances were observed. Abbreviations: N; nucleus, M; mitochondria. Scale bars: 1 μm. *p < 0.05; **p < 0.01; ***p < 0.001; and ****p < 0.0001

Addition of an unsaturated fatty acid can alleviate the membrane-rigidifying effects of PA in AdipoR2-deficient cells [19]. Here, HEK293 AdipoR2 siRNA- and non-targeted siRNA-treated cells were cultivated with PA and/or the monounsaturated OA. Co-treatment with OA significantly decreased the frequency and/or severity of the membrane defects in both NT and AdipoR2 siRNA-treated cells (Fig. 5A-C). In particular, the frequency of closely apposed membranes in the NT siRNA-treated cells was reduced while their severity (instances per cell) was reduced in the AdipoR2 siRNA-treated cells co-cultivated with PA + OA rather than with PA alone (Fig. 5E). For the NEBs and mitochondrial deformations, co-treatment with OA reduced their frequency in both control and AdipoR2-deficient cells in comparison to the PA alone treatment (Fig. 5F, G). Samples treated with OA alone showed no membrane morphology defects suggesting that it is well tolerated (Figs. 5D and E-G). The dose-dependent effects of PA and protection by OA in NT or AdipoR2 siRNA-treated HEK293 cells were replicated in an independent set of experiments, confirming their robustness; in particular, both the frequency and severity of closely apposed membranes was reduced in AdipoR2 siRNA-treated cells cultivated in PA + OA compared to PA alone in this repeat experiment (Figure S2A-C).

The protective effects of OA are consistent with membrane rigidification being the main mechanism for the observed PA-induced membrane defects. Earlier studies have shown that addition of PA to mammalian cells causes membrane rigidification [40–42], and we previously verified this in HEK293 and other cell types using fluorescence recovery after photobleaching (FRAP) and Laurdan dye assays [11, 16, 19]. Published lipidomic studies of HEK293 cells treated with/without the same AdipoR2 siRNA used here also showed that PA treatment results in an excess of saturated fatty acids in phospholipids and that the inclusion of OA counters the rigidifying effects of PA while normalizing the fatty acids composition in phospholipids [11, 16]. It is also possible that OA facilitates the sequestering of PA into lipid droplets, as has been documented in cultured cells and mouse embryonic fibroblasts [43], and in this way limits its effect on membrane rigidification.

### HAP1 AdipoR2 knockout cells showed similar cellular deformations

**Fig. 6:**
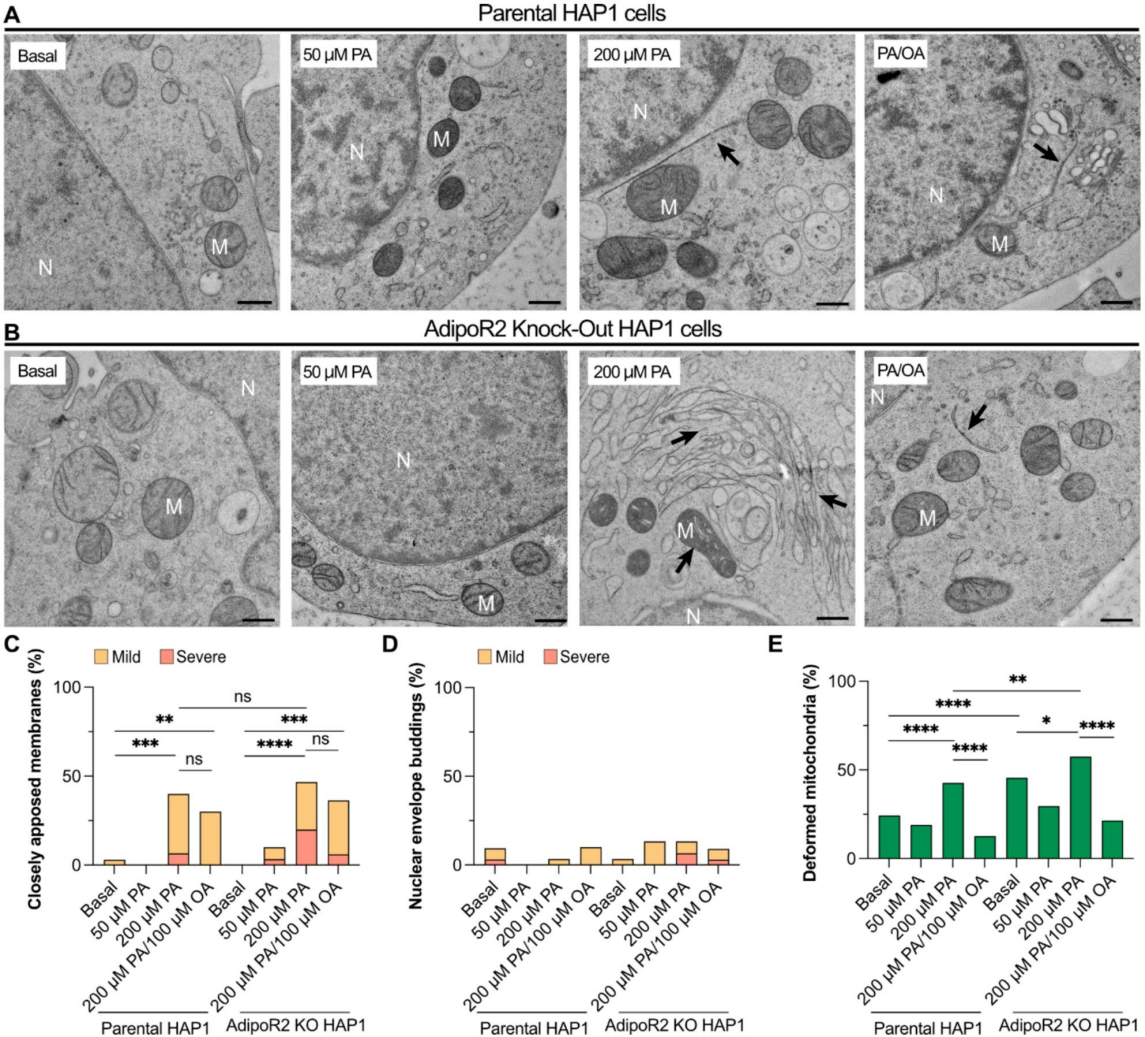
HAP1 cells exhibit similar PA-induced morphological deformations. Parental and AdipoR2 KO HAP1 cells were grown under 0 µM PA (basal), 50 µM PA, 200 µM PA and 200 µM PA/100 µM OA. Random electron micrographs of at least 30 cells were imaged and quantified for the three examined cellular deformations. **(A-B)** Representative images from the parental and AdipoR2 KO HAP1 cells for each treatment. Black arrows point at closely apposed membranes and deformed mitochondria. **(C-E)** Quantifications of closely apposed membranes, nuclear envelope buds and deformed mitochondria for each treated group (*n* = 30–33). In these cells, there was only a limited number of nuclear buds observed. OA was able to reduce the frequency of deformed mitochondria and alleviate the severity of the closely apposed membranes. The closely apposed membranes and NEB phenotypes were further categorized as “mild” when less than three instances were observed in one cell section or as “severe” when three or more instances were observed. Abbreviations: N; nucleus, M; mitochondria. Scale bars: 500 nm. *p < 0.05; **p < 0.01; ***p < 0.001; and ****p < 0.0001

To investigate whether the PA-induced membrane phenotypes occur in other cell types, parental HAP1 (a near-haploid cell line derived from a patient with chronic myeloid leukemia [44]) and HAP1 AdipoR2 knockout cells were treated with 0 µM PA (basal), 50 µM PA, 200 µM PA and 200 µM PA/100 µM OA.(Fig. 6A-B). Closely apposed membranes showed a PA dose-dependent increase in frequency in control and AdipoR2 KO cells, with the frequency and severity being highest for the AdipoR2 KO cells; OA could only partially but not significantly prevent these events (Fig. 6C). In contrast to the HEK293 cells, HAP1 cells did not show many NEBs in any of the tested conditions, with only 3 to 4 NEBs in 5–10% of cells in all treatments (Fig. 6D). Mitochondrial defects in parental HAP1 were increased in 200 µM PA, and this was prevented by co-treatment with OA (Fig. 6E). AdipoR2 KO cells in basal media already exhibited a high frequency of deformed mitochondria that was further increased by 200 μM PA and reduced by cocultivation with OA **(**Fig. 6E). These findings were reproduced in a separate set of experiments that confirmed their robustness (Figure S2D-F). To summarize, HAP1 cells are more resistant to PA-induced membrane defects than HEK293 cells but show similar trends especially with respect to the formation of closely apposed membranes, with AdipoR2 KO cells being most sensitive. Long-term cultivation of the HAP1 AdipoR2 KO line may have allowed them to adapt to AdipoR2-deficiency, which is not the case for AdipoR2 siRNA-treated HEK293 cells, and this may explain their weaker PA-induced phenotypes.

### Closely apposed membranes correlate with protein trafficking defects

As noted earlier, the mitochondria of cells exposed to PA or AdipoR2-depleted are enlarged, which is interesting given that mitochondrial size affects the autophagic process [45, 46] and that exogenous PA increases mitophagy in a dose-dependent manner, a process regulated by the protein PINK1 [47]. Under normal conditions, PINK1 translocates to healthy mitochondria where it is degraded. PINK1 is however unable to penetrate the outer membrane of defective mitochondria where it accumulates and recruits the PARKIN protein that initiates the mitophagy process [48]. Though an increase in PINK1 has been documented in PA-treated cells [49], the exact sub-cellular location of the protein has not been reported.

**Fig. 7:**
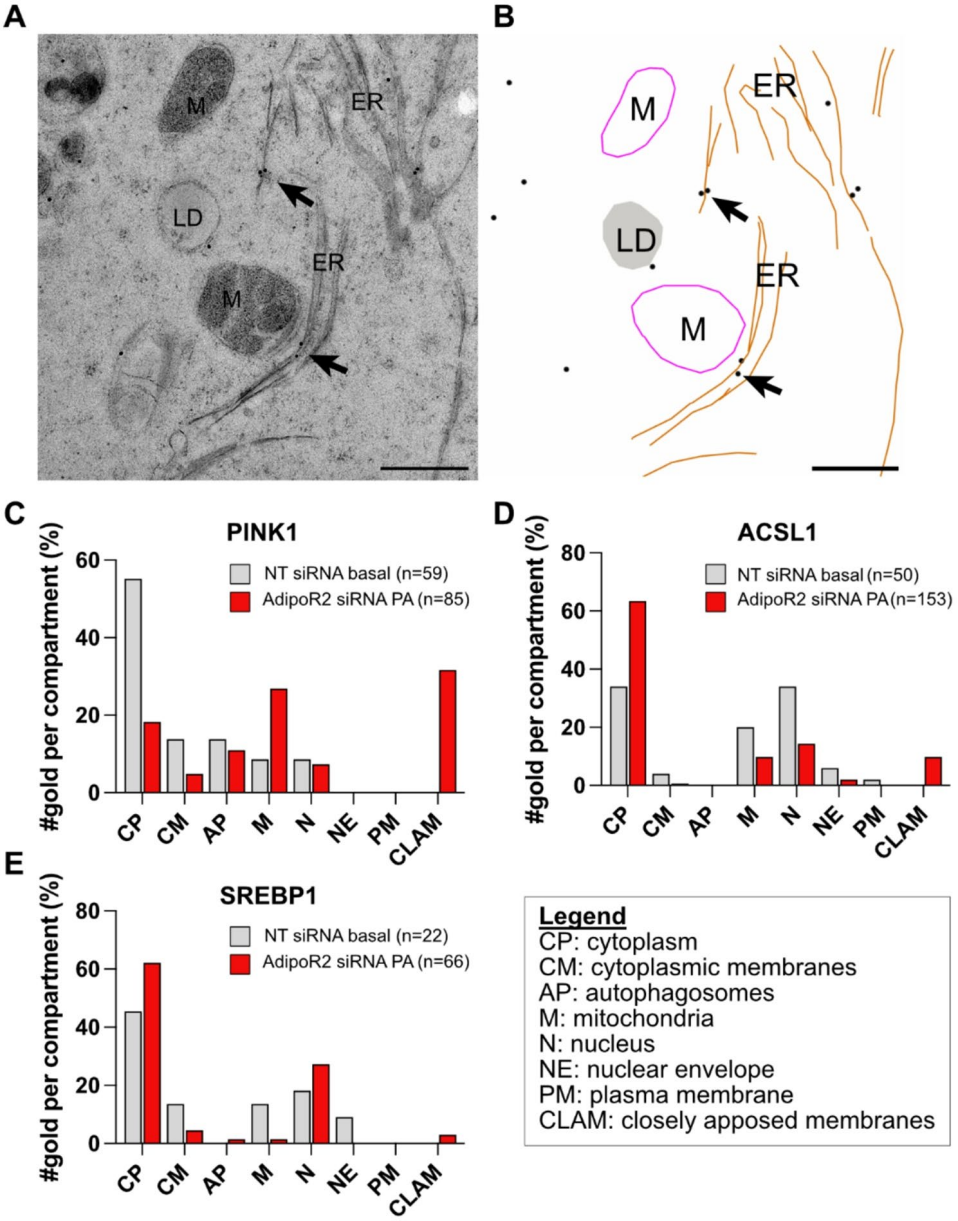
Both PINK1 and ACSL1 proteins are enriched on closely apposed membranes compared to normal ER.** (A)** Representative image of the closely apposed ER with PINK1 immuno-gold particles bound to it and **(B)** a model of the image for a clearer visualization of the gold particles. **(C-E)** Immuno-EM assays allowed quantification of PINK1, ACSL1 and SREBP1 localization among 8 different cellular compartments. Number of gold particles per examined cellular compartment for the NT siRNA-treated cells in basal conditions cells and AdipoR2 siRNA-treated cells challenged with PA. Both the PINK1 and ACSL1 proteins had a higher frequency on the closely apposed membranes when compared to normal ER. Abbreviations: CP; cytoplasm, N; nucleus, CM; cytoplasmic membranes, CLAM; closely apposed membranes, M; mitochondria, NE; nuclear envelope, AP; autophagosomes, PM; plasma membrane

Could the membrane defects in AdipoR2 siRNA-treated cells challenged with PA impact mitophagy by disrupting PINK1 trafficking? To explore this hypothesis, we used immuno-EM to localize PINK1, ACSL1 and SREBP1 among 8 distinct intracellular compartments. ACSL1 and SREBP1 were chosen because they participate in lipid biogenesis/metabolism and localize to the ER at some point during their trafficking [50, 51]. The number of gold particles per cellular compartment was counted and plotted against the area (nm^2^) or length (nm) of the membrane structure. We found that PINK1 and ACSL1, but not SREBP1, are enriched on closely apposed membranes of AdipoR2 siRNA-treated cells grown in the presence of PA (Fig. 7). We conclude that the closely apposed membranes likely disrupt the intracellular trafficking of some but not all proteins.

## Discussion

Previous studies have described some of the dramatic effects caused by excess SFAs in phospholipids including defects in vesicle formation, protein transport and receptor signaling as well as ER stress, ROS accumulation and activation of the apoptotic pathway [3, 20, 21, 52]. Several proteins that sense and rectify membrane defects in animal cells have been identified [6], including AdipoR2 that specifically responds to membrane rigidification [11, 19]. Though it is already well known that PA can cause many cellular defects, few studies have described these at the ultrastructural level [19, 22, 53]. Here, we quantified morphological abnormalities caused by PA and AdipoR2 deficiency in two model cell lines, namely HEK293 and HAP1 cells, and our observations were collected with the use of electron microscopy and immuno-EM assays. We found that three different membranous compartments were most affected by PA treatment and worsened in AdipoR2-deficient cells: the ER, the nuclear envelope and mitochondria. Note that an interesting difference was found between the two cell line models: HAP1 cells are more resistant to PA-induced membrane defects than HEK293 cells but show similar trends especially with respect to the formation of closely apposed membranes, with AdipoR2 KO cells being most sensitive. Long-term cultivation of the HAP1 AdipoR2 KO line may have allowed them to adapt to AdipoR2-deficiency, as observed in several other gene knockout models [54], and this may explain their weaker PA-induced phenotypes. Such adaptation may also explain why the AdipoR2 knockout mouse is generally healthy in spite of a pronounced excess in saturated fatty acids in its phospholipids [15, 55], except for a severe spermatogenesis defect [25].

### Closely apposed membranes and mitochondrial deformations

One of the most striking defects in PA-treated cells was the formation of closely apposed membranes in the cytoplasm that appeared as straight lines in electron micrographs. These lines were often long, spanning almost the whole cell cross section, though occasionally short, and accumulated in specific areas in the cell. The ER is a dynamic structure that can adopt a variety of shapes, including stacked cisterna when ER proteins are overexpressed [56], or spirals of tightly wound membranes that may appear as straight lines in flattened primary cells lacking AdipoR2 and that may be homologous to the ones observed in the present study [22]. Here, when HEK293 cells were immuno-stained for the mitophagy marker PINK1, we unexpectedly found that it accumulated on these closely apposed membranes to levels much higher than on control ER membranes. Under normal conditions, PINK1 associates with the outer membrane of healthy mitochondria, then imported into the inner membrane and degraded by the protein PARL. PINK1 is however unable to penetrate the membrane of defective mitochondria and thus accumulates on their surface to recruit the PARKIN protein that initiates mitophagy [49, 57]. A previous study reported that PA has a dual effect on the expression of PINK1: low PA concentrations (0.3 mM) led to increased PINK1 levels whereas higher concentrations (0.5 mM) had the opposite effect [49]. As these levels were detected through western blot assays, the specific localization of PINK1 in the PA-treated cells was unknown. Although PINK1 does not typically associate with the ER [58], it can sometimes associate with ER tubules [59] and the ER may have a role in regulating PINK1 dynamics [60]; the present study is consistent with this idea. Specifically, we show here that higher PA concentrations increased the frequency of closely apposed membranes that accumulate PINK1; this suggests that PINK1 associates with the ER in the presence of PA, reducing its ability to decorate defective mitochondria and allowing them to avoid autophagy and thus persist.

Challenging cultivated cells with palmitic acid is well known to cause mitochondrial defects including excess ROS production, reduced beta-oxidation and changes in morphology [61–64]. Here, we found that exogenous PA, especially in AdipoR2 deficient cells, causes the deformation of mitochondrial cristae into swollen structures. Similar defects have been observed under conditions of calcium (Ca^2+^) overload in cardiac mitochondria from guinea pig hearts [65]. In that study, a double role of mitochondrial Ca^2+^ was noted: low concentrations are essential for optimal rates of ATP production, but higher levels lead to cristae network disruption and an eventual loss of mitochondrial function. A different study also showed that PA-treated cells have elevated levels of cytosolic Ca^2+^ [66]. This effect was followed by depletion of ER Ca^2+^, and loss in mitochondrial membrane potential accompanied by a release of cytochrome c and activation of apoptotic pathways. Speculatively, the mitochondrial deformations that we detected could be a result of a PA-induced changes in concentration of cytosolic Ca^2+^, combined with impaired mitophagy due to PINK1 accumulation in the closely apposed ER membranes. Alternatively, the mitochondrial morphology changes may reflect an adaptation to nutrient availability in the presence of PA, as observed in the presence of e.g. galactose [67]. In any case, the mitochondrial defects were enhanced in AdipoR2-deficient cells, which is consistent with the previously reported reduced mitochondrial respiration in HEK293 cell lacking AdipoR2 [13].

### Nuclear envelope buds can form during cellular stress response

A third striking ultrastructural defect that we observed was the formation of blebbings (variably sized and shaped membrane-bound structures) located between the outer and inner nuclear envelopes. Previous studies have observed similar blebbings, sometimes referred to as nuclear envelope budding (NEBs) or other names [36, 68–72]. These NEBs may transport material, such as protein aggregates, through the nuclear envelope as an alternative route to the nuclear pore complex pathway [36, 71]. NEBs are more frequent in stressed cells, for example as a result of heat shock, or exposure to arsenite, hydrogen peroxide or proteasome inhibition [36]. In AdipoR2-deficient HEK293 cells, NEBs were frequent and may be taken as further evidence that these cells are under stress. In contrast, the HAP1 AdipoR2 KO cells showed fewer NEBs, even under PA treatment, suggesting better PA tolerance.

## Conclusions

Our main findings are that PA treatment causes three main cellular deformations, with the closely apposed membranes being the most dose-dependent and a potential cause of protein localization defects, as evidenced by their accumulation of PINK1 and ACSL1 proteins. Furthermore, this work confirms the important role of AdipoR2 in membrane homeostasis since cells lacking AdipoR2 showed membrane defects even in the absence of PA, which were further enhanced by PA treatment.

## Supplementary Information

Supplementary Material 1.

## Data Availability

No datasets were generated or analysed during the current study.

## Abbreviations

AdipoR2: Adiponectin receptor 2
ATCC: American Type Culture Collection
FA: Fatty Acid
FBS: Fetal Bovine Serum
INM: Inner Nuclear Membrane
NEB: Nuclear Envelope Budding
OA: Oleic Acid
ONM: Outer Nuclear Membrane
PA: Palmitic Acid
PFA: Paraformaldehyde
SFA: Saturated Fatty Acids
UFA: Unsaturated Fatty Acid
UA: Uranyl Acetate
