# Supplementary material for: Electron microscopy reveals saturated fatty acid-induced membrane defects in AdipoR2-depleted cells
Source: Lipids Health Dis. 2025 Dec 1;24:375. doi: 10.1186/s12944-025-02804-2 (PMC12670739; doi:10.1186/s12944-025-02804-2)
Supplement: Supplementary file 1 — Supplementary Material 1. [file 12944_2025_2804_MOESM1_ESM.pdf]

SUPPLEMENTARY MATERIAL

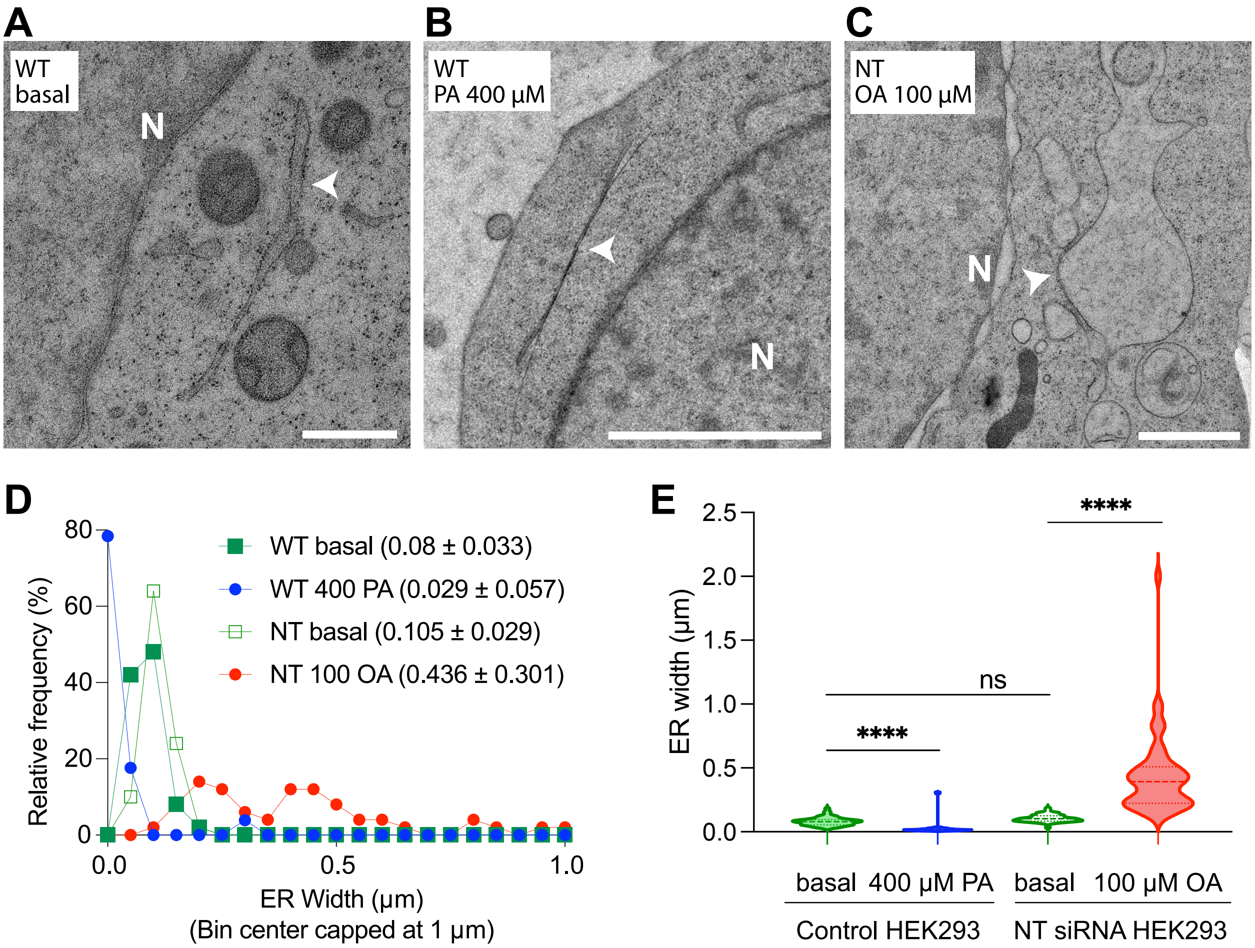

Figure S1. Measurement of ER width for the categorization of membranes into closely apposed membranes or swollen ER. The width of ER membranes from control (WT) HEK293 cells on basal conditions or 400 μM of PA and NT siRNA cells on basal conditions or 100 μM of OA were measured for the distinction between closely apposed membranes, normal ER, and swollen ER. **A-C.** Representative images of normal ER (A), closely apposed membranes (B), and swollen ER (C). White arrowheads point at the ER membranes. **D-E.** Width measurements for each cell type and condition examined displayed as frequency distributions with mean and standard deviations stated in parentheses (D) or violin plots (E). There is a distinct and significant difference between the width of normal ER, closely apposed membranes, and swollen ER. Abbreviations: N; nucleus. Scale bars: A; 2 μm, B; 500 nm, C; 1 μm. *$p<0.05$; **$p<0.01$; ***$p<0.001$; and ****$p<0.0001$.

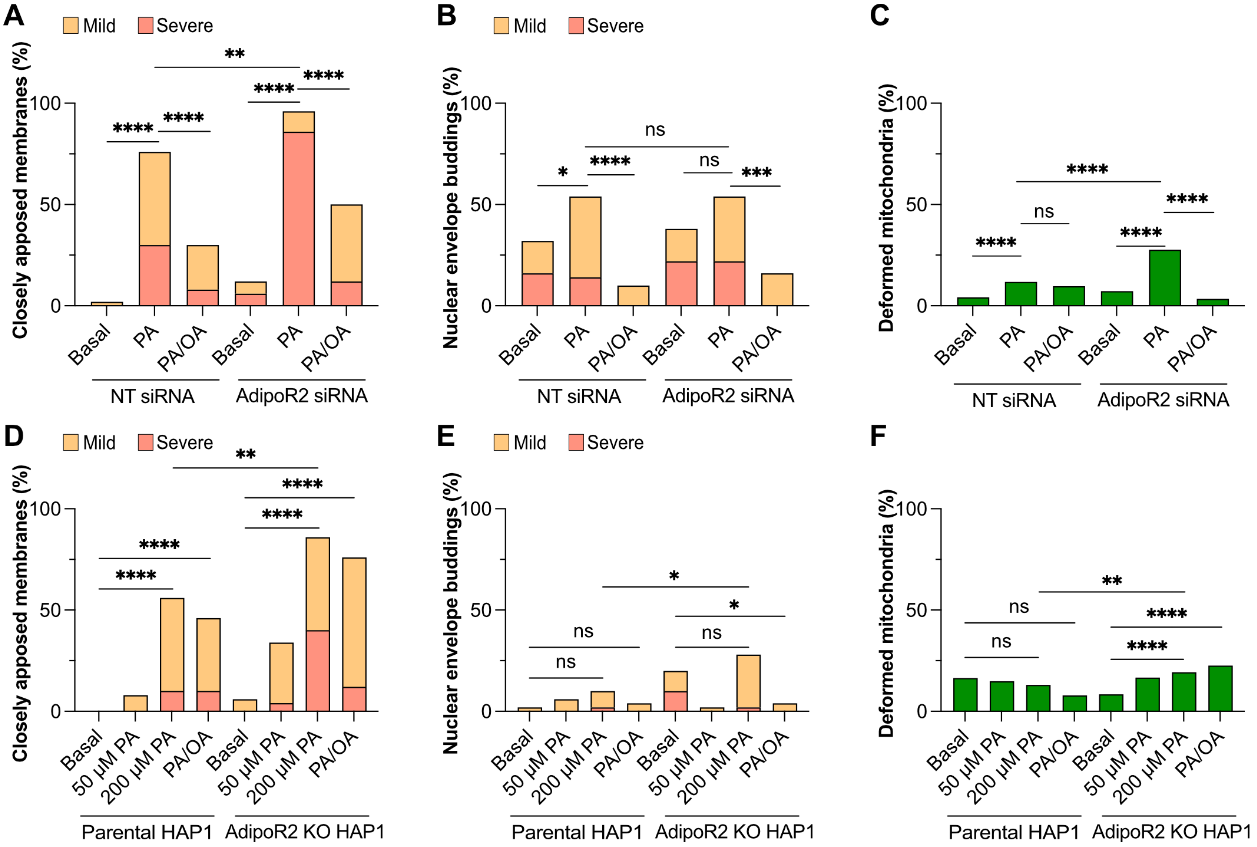

Figure S2. Duplicate experiments of the main findings in HEK293 and HAP1 cells. (**A-C**) OA rescue experiment in NT or AdipoR2 siRNA HEK293 cells (duplicate experiment of Figure 4). (**D-F**) HAP1 cells experiment with two different PA concentrations and co- treatment with OA (duplicate experiment of Figure 5). *p<0.05; ***p<0.001; and ****p<0.0001.
